# From cancer to rejuvenation: incomplete regeneration as the missing link (part II: rejuvenation circle)

**DOI:** 10.2144/fsoa-2020-0085

**Published:** 2020-06-30

**Authors:** Mamuka G Baramiya, Eugene Baranov, Irina Saburina, Lev Salnikov

**Affiliations:** 1 AntiCancer, Inc., San Diego, CA 92111, USA; 2 Institute of General Pathology & Pathophysiology, Moscow, Russia; 3 Russian Medical Academy of Continuous Professional Education, Moscow, Russia; 4 Department of Computer Science, Metropolitan College, Boston University, USA

**Keywords:** aging, carcinogenesis, epigenetic, immunological tolerance, immunoprivileged sites, looped ontogenesis, rejuvenation, remorphogenesis, reontogenesis, senescence

## Abstract

In the first part of our study, we substantiated that the embryonic reontogenesis and malignant growth (disintegrating growth) pathways are the same, but occur at different stages of ontogenesis, this mechanism is carried out in opposite directions. Cancer has been shown to be epigenetic-blocked redifferentiation and unfinished somatic embryogenesis. We formulated that only this approach of aging elimination has real prospects for a future that is fraught with cancer, as we will be able to convert this risk into a rejuvenation process through the continuous cycling of cell dedifferentiation–differentiation processes (permanent remorphogenesis). Here, we continue to develop the idea of looped ontogenesis and formulate the concept of the rejuvenation circle.

## Lay abstract

Here, we continue to develop the idea of looped ontogenesis and formulate the concept of the rejuvenation circle. We also briefly consider the issue of the applicability of our concept to postmitotic cells, which is of fundamental importance for the rejuvenation of the body as a whole and outline some areas of practical implementation of our proposed concept.

So I do not know how to tell you that I only can see that cancer become a friend to protect or even create a situation in our body that we can live longer. I am absolutely certain that time will change. But still mankind's do have ideas that they have the power to influence all nature and put themselves above God. Illness has – like everything in all nature – an evolutionary process. Cancer is one of the last steps in this process and accumulate especially at the end of every aion (+/− 2000 years). It is the cosmic sign, that the apocalypse (a total change of mind) is coming. As longer as people do not want to see – see it, to feel it, to smell it – deep inside they will go on with enemy-thinking.

S.W. Bok, May 21, 1997.

If everything regenerated, there would be no death.

Richard J Goss, Principles of Regeneration [1].

Currently, there are different views on the nature of aging and various methods of geroprotection in the fight against age-related diseases. Among them are the use of plasma factors (e.g., GDF11 and TIMP2), various variants of metabolic intervention (including rapalogs, metformin, resveratrol etc.), ablation of senescent cells (including senolytics) and the effects on telomerase. It should be noted that the main approaches are aimed at correcting the individual mechanisms associated with aging and age-related diseases. The same happens in oncology, although there is a wide variety and continuing improvement in the field of cancer treatment methods, they are all aimed at the killing of cancer cells. However, even with certain achievements, we are still far from radical results – victory over aging and cancer. In this article, we continue to elaborate the essence of our concept of the relationship between carcinogenesis and aging, which was described in part one [2].

## Tumor cells: enemies born as friends (short retrospection)

Aging is a process and a consequence of processes brought about by steadily increasing restriction of the self-renewal ability, limiting life expectancy and leading to an increase in the probability of death and, inevitable death resulting from the fading of functions, failure of the regulatory mechanisms, occurrence of endogenous disorders and increased susceptibility to exogenous factors [3]. In our opinion, one of the fundamental (systemic) flaws of gerontology is the idea of the existence of a special aging program and the search for the cause of aging, which states that if removed, aging can be eliminated. However, there is only one general program, a program of growth and development (ontogenesis), of which aging is an integral part. The essence of this program is the stabilization of multicellular integrity by submitting the purposes of the constituent parts (cells) to the purposes of the whole (tissues, organs and the body in entirety), through the epigenetic restriction of cell potencies in favor of perfecting (complicating) tissue specialization, for what we pay for with aging, all types of endogenous pathology and, as a result, mortality. From this, it follows that the ‘cause’ of aging is not some special mechanism but a program/order, which can be overridden only by implementing another program, a program, of permanent, unlimited, quantitative and qualitative full restoration of structures, functions and functional interconnections. In other words, the linear unidirectionality of ontogenesis, fatally leading to aging and death, can only be overcome with permanent reontogenesis, through the looping of this linearity. This does not require an application of any force against nature, because similar processes were invented by nature itself and because they work in practically immortal multicellular organisms, such as *Hydra vulgaris.* It is important to note that *Hydra* does not have cancer as a pathological process. In other words, a periodic return or ‘rollback’ to the blast state does not cause cancer (disintegrating growth, DG) in those types of immortal organisms.

It is the restoration of cells that restores tissues, organs and functions. aging at the whole-body level is predetermined by aging at the cellular level. Consequently, reontogenesis can be carried out only through cells that are capable to continuously, unlimitedly, quantitatively and qualitatively self-renew. This can be performed only by cells, for which senescence is not the final stage of development but only an intermediate one, that are capable of constantly ‘zeroing out’ the genetic and metabolic ‘burden’, restoring functions and functional interconnections. There are only two types of cells that satisfy these criteria. Cells of the first type are the non-aging totipotent or pluripotent cells of early embryogenesis, which, in addition, effectively get rid of even advanced glycation end products [4,5]. Cells of the second type are cells of precancerous tissues that, through spontaneous reprogramming (via the same regulators, Notch, Wnt, Hedgehog, OSKM and others), can reverse aging, but, at the same time, cause cancer [6,7]. It should be noted that the same happens as the result of using main cancer treatment methods (chemotherapy, antiangiogenic therapy, radio- and immunotherapy), wherein senescence and death of tumor cells give rise to a spontaneous *de novo* wave of reprogramming with the return of resistant forms of cancer [6], and for all types of induced reprogramming for rejuvenation purposes [8,9].

Conclusion. Any methods affecting both individual ‘aging’ mechanisms and age-related pathology will only alleviate the burden of aging, and these diseases slow down their progression but will eliminate neither aging nor age-related diseases. By eliminating aging, we will eliminate age-related diseases.

In our early article [10], the proposed concept was first formulated, and we summarized data demonstrating that the morphogenetic field of the embryonic environment reverses malignant phenotypes – ‘reprogramming’/’returning’ tumor cells back to normal cells ones. Based on this, we concluded that tumor cells do not lose their normal morphogenetic potencies, but are also capable normalizing, and even giving rise to, a normal whole organism. As it has been demonstrated in a number of studies [11–17], the ability of normalization is a characteristic not only of germ cell tumors but also other tumors, such as leukemia, melanoma, liver and breast cancer, nephroblastoma, medulloblastoma, glioblastoma and others. This indicates that tumor cells have the ability to ‘sense’ themselves and the environment through morphogenetic signaling – to tune and self-adjust – integrating into the normal morphogenesis. On the other hand, embryonic tissue transplants in non-immunoprivileged sites give rise to the existence of tumors. In this case, there is not an escape from the morphogenetic field as suggested by Needham [18] and Waddington [19], but there is an external blocking of the susceptibility of cells to morphogenetic signals. The data of Shvemberger [20] on the normalization of tumor cells (rhabdomyosarcoma, Ehrlich ascites carcinoma) in the non-embryonic zone of the eye anterior chamber, which has only immunoprivilege in common with the embryonic zone, allowed us to state that it is the immunoprivilege that provides this ability to ‘sense’ oneself and the environment, as well as how to be subject and object of normal morphogenesis. The immunoprivilege provided by trophoblast and deciduas [21], and the transformation of immune surveillance effectors (T cells, Natural Killer [NK] cells, T helper [Th] 1 cells, M1 macrophages, dendritic cells [DC]) at the mother–fetus interface into regulatory subtypes (T regulatory cells [reg], NK reg, Th2, M2 reg macrophages, DC reg), protects the embryo not only from rejection but also from becoming a tumor. For example, maternal uterine NK reg (as well as the rest of the regulatory subtypes) contributes to the implantation of the embryo by invading a trophoblast consisting of nondividing cells, placentation, spiral artery remodeling and active immunotolerance [22,23]. An important role in these processes belongs to trophoblast cells expressing human leukocyte antigen HLA-G and HLA-E isotypes of the major histocompatibility complex [24,25]. The same happens with the participation of the same isotypes [26–28] and mechanisms [29] in nonembryonic conditions. On the other hand, spontaneously reprogrammed cells that recapitulate the program of early ontogenesis (embryogenesis) are in direct contact with immunity effectors, which are transformed at the tumor–host interface into regulatory subtypes in the absence of a protective/shielding trophoblast (absence of immunoprivilege). This results in the blocking of the ‘susceptibility’ to morphogenetic signals, the distortion of a unified morphogenetic field, the reinforcement of a progressive autonomous status and the conversion of normal growth processes into malignant (disintegrating) growth.

It should be noted that the reprogrammed cells, as well as the embryos, still have some kind of protective ‘shield’ the fibrinoid layer, so-called Nitabuch striae, masking their antigenic structures [30], because the activity of enzymes contributing to the deposition of fibrin in these cells is significantly higher than in differentiated somatic cells [31]. However, activated macrophages have significantly more fibrinolytic activity when compared with non-activated ones and therefore, deprive these cells of this protective shield [32]. In addition, M_2_reg macrophages, like other regulatory subtypes of immune response effectors, promote growth processes. Once again, it is important to emphasize here that the transformation of immune surveillance effectors into regulatory subtypes at the mother–fetus and tumor–host interface is not immunosuppression, but only immunomodulation. This manifests one of the main morphogenetic functions of the immune surveillance system, maintaining cellular (tissue) homeostasis (morphostasis), which is carried out through maintaining the growth process in damaged tissues. In this process, the immune response to proliferating cells is carried out through the mechanism of active tolerance, when there is an immune response; however, it does not kill but supports. As others also pointed out, the main behavior of the immune system is determined by the rules governing cell survival and perpetuation and organismic homeostasis maintenance and the self-versus-non-self recognition paradigm is no longer the center of the immune system concerns [33]. This was described in more detail in the first part of the study [2].

It can be concluded that tumor cells at any stage of progression are able to revert to normal ones subject to two conditions, namely, the presence of morphogenetic signaling of the environment and the ability to ‘hear’ it and themselves, which is ensured by immune privilege or, in the of nonembryonic conditions, complete immunological tolerance toward them.

Thus, we formulate tolerance as a defense strategy from cancer, as it has been proposed by Medzhitov *et al.* for infectious pathology [34].

The cancer eradication paradigm on which all modern oncology is based plays a major role in the palliative care of patients. However, we are confident and firm that this paradigm will never eliminate the cause of cancer but will only reduce the probability of dying from cancer for some time and allow individuals to die from some other pathology. On the other hand, when one attempts to eradicate cancer, one terminates rejuvenation, because spontaneous reprogramming, which then turns into cancer, is an attempt by any living matter to self-renew. Full-scale reprogramming (spontaneous or induced) that never turns into cancer does not have a fully fledged biological alternative for simultaneously solving two main problems: eliminating both cancer and aging. They cannot be solved separately, because the solution to these problems is the same. Trying to eliminate aging, we will ‘call for’ cancer. Moreover, trying to eradicate cancer, we will still be destined to aging.

It has to be emphasized that only half measures lead to half results. The goal of ‘truncated’ or partial reprogramming protocols is clear: to avoid the formation of a tumor. However, this approach does not and most likely will not lead to a radical elimination of aging, since it does not allow ‘to erase’ the critical hallmarks of aging. Furthermore, as noted, “*failure to erase critical hallmarks of aging may lead to refractory populations of cells and cellular senescence*” [35].

## One-way ticket: the death road or some thoughts about ontogenesis

**Figure 1.**
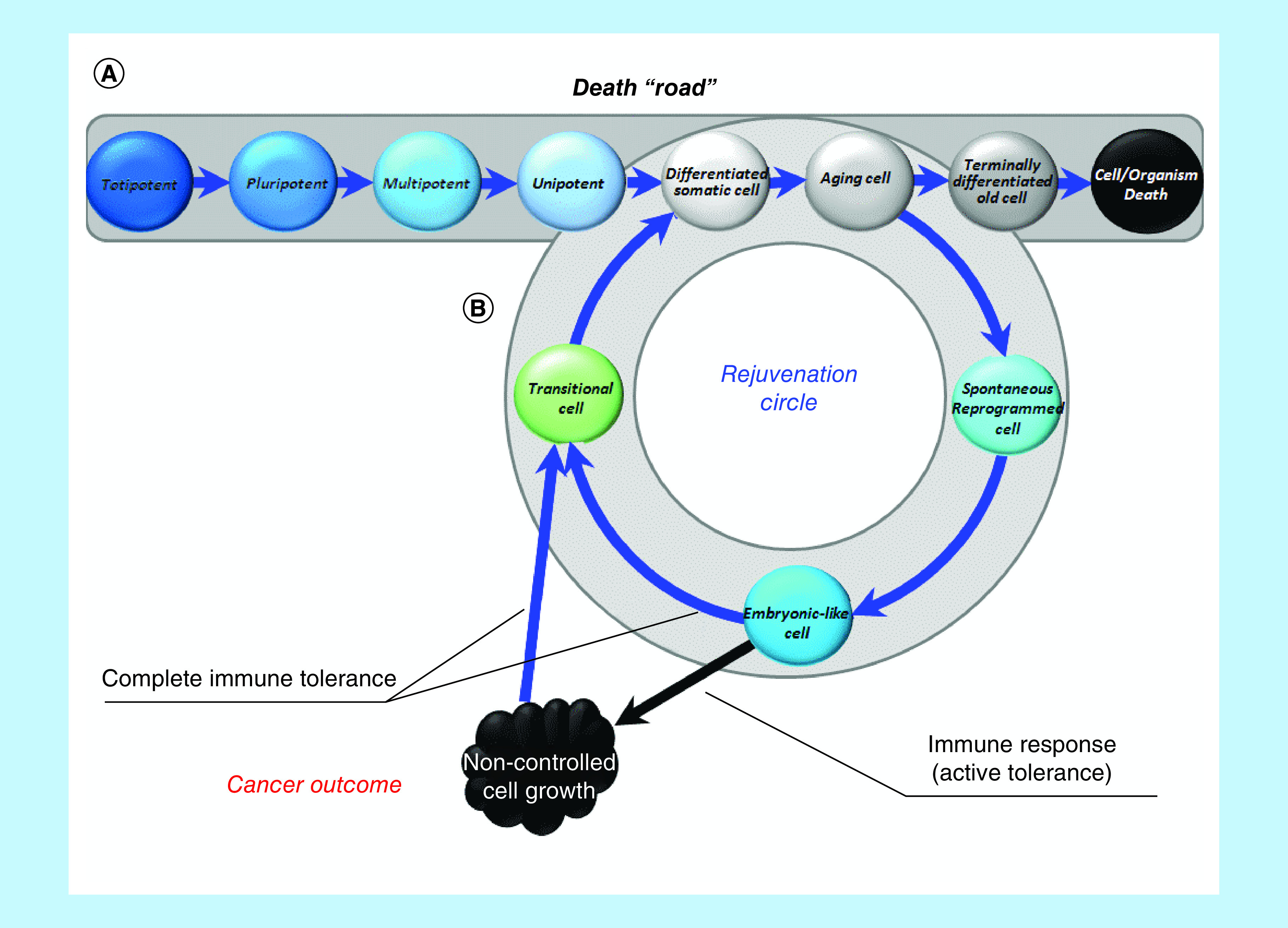
Linear (classical) ontogenesis or ‘death road’. Linear ontogenesis **(A)**. Looping ontogenesis or rejuvenation circle **(B)**.

Each of us is already born with a one-way ticket to the graveyard (Figure 1A). Our path begins with the merging of two differentiated ancestors – germ cells. After approximately 3 days, we are just a few totipotent cells that can divide (renew) without limitation, giving rise to any of the tissues of which we will later consist. Starting from day 4, an important event takes place: we get armor, which will be needed in order to survive in the womb, where we are just a transplant (which, if everything goes according to the scenario, will be rejected at the right time through birth).

The first differentiating event in ontogenesis begins with the formation of an armor called trophoblast. Trophoblast is the first differentiated specialized tissue in our life that will accompany us until birth. It is its layer, called syncytiotrophoblast (and then the deciduas), consisting of nondividing multinuclear cells and encounters the invasion of the mother's immune system effectors, that protects us from many unfavorable outcomes, such as death, premature birth, transformation into teratoma/teratocarcinoma.

At this moment, everything, which later becomes us, is pluripotent, such that it can still proliferate without limit and can give rise to everything, but also an ‘armor’. Covered with that ‘armor’, we begin the struggle for our existence, engrafting ourselves into the uterus (implantation). Having finally implanted (7–8 days), we are divided into three parts (three germ layers), while remaining unified, but moving at the same time toward three different directions (gastrulation). As soon as the separation has occurred, each of those layers (ectoderm, mesoderm and endoderm) can become only that tissue / organ, the formation of which is determined only by its path. Currently, we already lose pluripotency and before becoming multipotent. Gastrulation (the mesoderm is formed later than all) is completed by approximately day 35, and organogenesis begins, during which multipotency is gradually lost. By the end of organogenesis (end of 4 months), we consist of completely differentiated tissues. We then grow/mature, and by the due date, when the antigenic landscape of our armor (which has long time become a placenta) changes, we get rejected as a classic transplant. Afterward, we are already incapable of unlimited self-renewal (only partially recovering losses) and with a one-way ticket in hand, begin our unidirectional path to the graveyard. For the Hayflick clock is already ticking and a certain number of ‘ticks' are measured out, after which the spring is critically shortened and the clock stands up. By this time, the critically senescent cells cease to reproduce themselves. Under normal conditions, the spring can no longer be started. It's like a guardian whose duty does not allow critically aged cells to multiply. We continue to live and accumulate waste and various by-products and it becomes more difficult to get rid of them.

This is how differentiation (and specialization of cells and tissues), carried out through epigenetic drift, which determines the unidirectional process (from totipotency to differentiation), ensures our existence in the form of an integrated highly specialized whole organism. However, this also inevitably results in a narrowing of the spectrum of adaptive properties and a decrease in the reparative and regenerative potential, as well as both quantitative and qualitative restoration ability of the lost structures by new fully functional ones. It also leads to deterioration in the ability to dispose the metabolic waste that in turn results in the decline of growth–reparative potential and relative adaptive rigidity of differentiated structures. Indeed, in order to restore large volumes of damage, more freedom to operate is needed than this can be provided in the framework of a relatively strict determination and the level of differentiation determined by it.

This is the essence of senescence. Summing up for individual tissues and organs, it ultimately leads to the aging of the whole organism (because aging of the whole is the sum, though not simple, of senescent parts). Initially, there are no visible changes in the appearance; however, starting at some point, it begins to change, and the state of the flesh also worsens. We are no longer able to adequately maintain an acceptable level of health.

What can be done? Live ‘slower’, get rid of senescent (and at the same time quiescent) cells, maintain a healthy lifestyle and live on a schedule, watch every piece eaten and drunk? This may postpone the manifestation (and maybe even initialization) of diseases. Perhaps they will not all pile up at once, and the sign ‘the end’ will also move little further away, but no more. It can prolong and mitigate the aging itself, and this is the maximum you can hope for. Look at the photos of centenarians. Do we just agree to fade in this way? In the end, is the difference so large? What class do we travel if, after all, it is one-way ticket? Are we satisfied with waiting at the death reception hall, even if it is huge and decorated with many various exciting and beautiful things? In fact, these are the only services included in the one-way ticket.

Having created technologies that affect separate mechanisms, genes, pathways, one will eliminate neither aging nor disease – but will only ease their course and delay their onset. There are no, and cannot be, any youthful factors from a young environment (such as a blood plasma) or rejuvenating mixes, they are just palliatives, temporary ‘cosmetic’ products based on current and often conflicting trends. These are just modifications of the earlier approaches. Only by creating the technology to eliminate aging can we eliminate both aging and the age-related diseases. This is impossible to accomplish in principle by acting on individual mechanisms, genes and pathways, because aging is an integral part of a development program (ontogenesis) and because only a program can override a program, not a separate mechanism. This program does not need to be created artificially, because it is inborn and was created by millions of years of evolution and can operate in us as it already operates in other immortal organisms. Initialization of that kind a program will radically solve the problem of aging.

The intercellular environment is created by cells; therefore, an aging environment is created by senescent cells but not *vice versa*. The aging environment only exacerbates the cellular senescence. No matter how you change an intercellular environment to an ideally younger one, the cell will still age and die (which was demonstrated by Hayflick). Therefore, the only option for a radical biological solution to the aging problem is radical reprogramming. It is the cancer that starts as a spontaneous reprogramming (as rejuvenation attempt) and only then becomes cancer. Knowing the cause for such a transformation, one will know its essence and then will be able to direct the processes toward the rejuvenation, preventing their transformation into cancer. One can turn a mortal enemy into a friend that can eliminate both cancer and aging. This is exactly what makes the proposed concept unique.

A complex specialized multicellular organism at the cellular level cannot but differentiate and, therefore, must age to remain an integrated specialized structure. An alternative is the transformation into one large disorganized structure (tumor) of ageless, pluripotent, immortal cells. However, we are actually capable not age at the level of the whole organism, because DG is an unlimited (NB!) expansion of the potencies of the constituent parts within the whole. It is this limitlessness that determines the disintegrating / malignant nature of tumor growth. However, we can escape both cancer and aging if the recapitulation of the embryonic program will only be temporary and cyclical in nature, not depriving cells the ability to differentiate again and making both senescence and rejuvenation not final, but only intermediate stages (Figure 1B). That is the essence of the proposed solution, which can be called as permanent reontogenesis or a rejuvenation circle. Multipotent tissue-specific progenitor cell status will be sufficient only for normal tissue-specific regeneration, but may be not sufficient for complete self-renewal, at least because these cells wouldn't be able to cope with advanced glycation end products and demonstrate a stable decrease in epigenetic age. That is why we have included ‘embryo-like cells' in the rejuvenation circle, suggesting that only this type of cell status can provide a solution to the problem of complete quantitative and qualitative self-renewal of tissues and functions.

## Permanent reontogenesis or rejuvenation circle

Any primary cause has many consequences, each affecting this cause through various feedbacks, and they themselves become the cause of many consequences. This complex polyphony consists of many notes (genes and factors encoded by them) and melodic lines (signaling pathways). It is necessary to take into consideration various transcription options from alternative promoters to post-transcriptional regulation, with the formation of functionally different isoforms that regulate various additional signaling pathways, creating a truly cosmic ‘cobweb’ of relationships and interdependencies with the cosmic variety of options through positive and negative feedbacks. In that orchestra, each note and melody line are aware where to join and where to fade, when and how to combine with others, when to upregulate and when to downregulate one's activity. All cells, where this whole orchestra plays, hear themselves and their environment through self-tuning and they know their path, becoming an integrated unit in the tissues they form. As a result, cells create a sophisticated web of morphogenesis under conditions of immune privilege, when nothing hampers them.

It is namely the incomplete/fragmented morphogenesis in the absence of this immune privilege is a cancer, but not mutations that are secondary, being only an epiphenomenon/by-product [36–38] of this fragmentation and non-blocked reverse of ontogenesis. As other authors noted, only a few mutations are found in the majority of tumors, whereas the majority of mutations are observed in less than 5% of tumors [39]. The above is consistent with the tissue organization field theory proposed by Sonnenschein and Soto [40], which, in our opinion, can also be called the theory of the morphogenetic field (an area of an embryo that responds as a coordinated unit to embryonic induction through the formation of multiple and differentiating – in a determined direction – anatomical structures). The same researchers were right, pointing out the following: “*Now, if neoplasia, as posited by the somatic mutation theory, is due to the accumulation of multiple mutations, how can cells derived from these tumors revert to behave as normal cells? It is statistically unlikely that random reverse mutational events that erase the previous mutations would be responsible for this reversal*” [41].

The above data on the normalization of tumor cells, as well as the fact that the administration of induced pluripotent stem cells into the embryo with a mutation leading to the downregulation of p53 with the simultaneous upregulation of growth/proliferative signaling pathways typical for embryonic stem cells (ESCs, which, when introduced into an extraembryonic environment, would highly likely lead to the formation of a tumor), resulted in to the development of a normal mouse [42]. This allows us once again conclude that where the morphogenetic module is susceptible to embryonic induction, we have morphogenesis, and where is not, we have carcinogenesis. This is precisely the essence of carcinogenesis.

Other authors also correctly noted in the article, which summarized the identical fundamental properties of embryonic and cancer cells [43], placing cancer cells into a ‘normal’ embryonic morphogenetic field, can reverse the malignant phenotype or ‘reprogram’ the cancer into normal cells. Therefore, it is not surprising that cancer cells exposed to specific embryonic morphogenetic fields undergo significant modifications, ultimately leading to complete phenotypic reversion. This is also supported by other authors [44,45]. It is important to note that some studies validate the persistence of morphogenetic fields throughout whole postnatal ontogenesis: these fields control histogenesis and organogenesis in embryogenesis, as well as maintaining and regenerating tissues throughout the entire postnatal ontogenesis [46–48]. In addition, it should be noted that the generation of a morphogenetic field in deficient tissues (partial hepatectomy) [48] is consistent with the fact that it is the early stages of embryogenesis, which generate oncostatic as well as differentiating properties, in that no effects can be seen after morphogenesis and organogenesis are complete [49,50].

Several misunderstandings occur when trying to present a whole picture of fundamental processes that coming from the fact that the dualistic function of genes, factors, their encoded and signaling pathways through which their actions, or their antagonistic bifunctionality, are not always taken into account [51]. This gives rise to seeming paradoxes and opposing conclusions.

As Eicher and Washburn correctly pointed out, while each gene is perfectly wild-type within its ‘own field’, they act as a deficient mutant when placed in a different environment [52]. As a consequence, the same gene can activate different pathways in different fields [53]. Hence the situation where the interpretation of any fact in a particular case seems to be correct, but when trying to build a whole picture on the basis of this interpretation, it turns out to be incorrect. That is why, for example, normal genes, the re-expression of which under non-embryonic conditions is associated with cancer, are incorrectly called oncogenes, although they are not the cause of cancer *per se*.

It was not an intention to review all possible types of the interaction of genes, their isoforms and signaling pathways, because the cosmic ‘web’ of relationships and interdependencies with the cosmic diversity of variants is practically boundless. Without knowing the whole complex multidimensional picture of all interactions, interconnections and functions in a living organism, it is impossible to intervene in one or another link and count on the desired effect. Based on some examples and reasoning, it has been demonstrated that living systems are able to self-adjust and materialize their inherited potential created over a billion years of evolution, under conditions where nothing interferes with them and without intrusion in complex occurring processes (no one controls embryogenesis, is it right?), where still little is known and where only an insignificant part will be known for long time. Much still remains unknown but, having rejected an enemy-thinking approach and accepting the principle of cooperation rather than interference, it would be possible to solve many problems.

Thus, the task is as follows: to create conditions equivalent to embryonic development in the postnatal period and, by achieving complete immunological tolerance to cells that restart the embryonic development program, to prevent the transformation of reontogenesis into carcinogenesis.

The rate of *P53* (localized on 17p13.1) mutations nearly accounts for 50% of human cancers [54]. However, it should be noted that *P53* is a classic recessive suppressor that requires a biallelic mutation to inactivate it [55], which is extremely rare. In addition, the vast majority (80%) of *P53* mutations are missense mutations, that is, when the substitution of one base is similar in its physicochemical properties to the wild-type, the tertiary amino acid structure is unchanged; therefore, the biological properties of the protein practically do not change. That is why *TP53* mutations do not strictly correlate at the individual level with the patient's clinical outcome [56], as it was also demonstrated by other authors. Moreover, 11 out of 24 types of cancers have shown significantly poorer survival rate with a high *P53* signature. No cancer variants have indicated poorer patient survival with a low *P53* signature [57]. When *P53* is not mutated, which is in approximately 50% of tumors, the upregulation of its negative inducer Mdm2, is mentioned. It is not always taken into account that normally the overexpression of Mdm2 activates blockade mechanisms of cyclin-dependent kinases [58]. Such interconnections support the balance between cell division/arrest cycle and dedifferentiation/differentiation, resulting not in tumor growth but in morphogenesis. The same happens in embryogenesis, where Mdm2 is critically required, and unrestrained *p53* activity during development causes early embryonic lethality, as occurs upon loss of the *p53*-negative regulators Mdm2 or Mdmx [59]. At the same time, as it is known, the overexpression of *P53* correlates with carcinogenesis and is an early event in the development of some human carcinomas [60–63].

Two orthologs of p53 members of the *P53* gene family are known, namely, tumor suppressors *P73* (localized in 1p36.33) and *P63* (3q27-29), which can compensate for the loss of *P53* [64] and which extremely rarely mutate (nature has developed reliable protection mechanisms for hundreds of thousands of years of evolution). All three members of this gene family function as transcription factors and regulate the expression of similar gene groups through direct binding to those sites, which are identified as p53-binding sites in the promoters. Transcriptional activation of these target genes leads to the induction of cell cycle arrest and apoptosis.

Full-length isoforms TAp73 regulates tumor angiogenesis by suppressing pro-angiogenic and pro-inflammatory cytokines. TAp73 activates p53-sensitive genes, such as *CDKN1A* (which encodes WAF1, also known as CIP1 and p21), *P53R2, PUMA* and *BAX*; therefore, like *P53*, it regulates growth arrest and cell death. In contrast, dominant negative ΔNp73 leads to stimulation of tumor growth and angiogenesis, inhibiting both TAp73- and p53-induced apoptosis. In turn, ΔNp73 is induced by TAp73 and p53, creating a dominant negative feedback loop [65,66].

Similarly, full-length TAp63 suppresses tumors, but ΔNp63 has oncogenic functions [67] also due to functional interplay between ΔNp63 and Δ133p53α [68]. However, ΔNp63α has been reported to inhibit cell invasion in cancer [69]. At the same time, ΔNp63α transcriptionally activates the E3 ligase HERC3, which mediates the ubiquitination of the *c-Myc* modulator MM1, making it the target of proteasome degradation, leading to cell cycle progression; therefore, *c-Myc* is depressed by the ΔNp63α factor, and MM1 knockdown prevents cell aging induced by a deficiency of either ΔNp63α or HERC3 [70,71].

It should be noted that not only the full-sized isoforms p73 and p63 play an important role in embryogenesis, normal development and implementation of physiological functions [68] but also ΔNp73 [72,73] through the expression of hTERT in telomerase-negative cells by blocking E2F-RB-mediated repression of the main promoter(s) hTERT [74] and ΔNp63 [75–77].

Antagonistic bifunctionality of *P53* is also carried out through its isoforms, the formation of which occurs through three possible mechanisms, namely, alternative splicing of pre-mRNA, initiation of transcription from alternative promoters and alternative initiation of protein translation. At present, 12 protein isoforms of p53 encoded by nine TP53 mRNA transcripts are known: (p53α, p53β, p53γ, Δ40p53α, Δ40p53β, Δ40p53γ, Δ133p53α, Δ133p53β, Δ133p53γ, Δ160p53α, Δ160p53β, Δ160p53γ). They are expressed jointly and work in coordination to regulate cellular responses. The p53 isoforms play an important role in normal embryogenesis and only in their ectopic expression, disrupting the balance between them and deregulating the signaling pathways leading to various disorders. For example, on the one hand, it is known that increased Δ40p53α expression is associated with carcinogenesis. On the other hand, with mucinous ovarian cancer, Δ40p53α expression is associated with improved recurrence-free survival, as well as functionally inactivates PTEN, which regulates insulin-like growth factor signal transduction to Akt, thus dysregulating IGF-1 signaling and consequentially promoting cellular senescence and reduced proliferation [78].

Elevated p53β and reduced Δ133p53α expression are a senescence-associated signature [79]. The restoration of Δ133p53α expression was sufficient to extend the replicative life span and delay in aging Hutchinson-Gilford progeria syndrome (HGPS) fibroblasts that exhibit low levels of Δ133p53α and high levels of p53β. Conversely, Δ133p53α depletion or p53β overexpression accelerated the onset of aging in proliferative Hutchinson-Gilford progeria syndrome fibroblasts [80]. In several cancer cell lines and normal fibroblasts, the p53β overexpression induces apoptosis and cell senescence via the upregulation of genes such as *BAX* and *p21* / miR34 in a p53-dependent manner. On the other hand, Δ133p53β stimulates the expression of key pluripotency factors like SOX2, OCT3/4 and NANOG [81], the expression of which is a key target for reprogramming in regenerative medicine.

The overexpression of Δ133p53α associated with carcinogenesis [82–85] has been reported in cholangiocarcinoma, cancer of the lungs, colon and ovaries and, has been associated with lower recurrence-free survival in patients with colorectal cancer. However, the expression of Δ133p53α has been associated with an improvement in recurrence-free survival and overall survival in advanced serous tissue of ovarian cancer with the *TP53* mutant gene. This suggests that it is impossible to attribute an absolute oncogenic or oncocompressive role to these isoforms, since their activity depends on the function of cells, the cellular context and the co-expressed drivers [78]. Other authors also point to this, noting that Δ133p53α may provide some protection against tumors [86].

It is known that Δ133p53α antagonizes p53 apoptotic activity and is an evolutionarily conservative pro-survival factor in DNA damage, which prevents apoptosis and promotes DNA double stand break repair to inhibit cell aging by enhancing the transcription of the repair genes *rad51, lig4 and rad52* by binding to a p53-sensitive element in their promoters [87]. Co-expression of Δ133p53 and the p53-p73 ortholog significantly stimulates DNA double stand break repair mechanisms, including all three pathways, namely, homologous recombination, non-homologous end joining and single-strand annealing [88]. At an early stage after damage, full-length p53(FLp53) is quickly accumulated in cells containing severe DNA damage, which inhibits DNA repair and results in cells undergoing apoptosis. In cells with smaller and fixable DNA damage, p53 is accumulated at a relatively low level in such a way to activate the transcription of target genes, including MDM2 and Δ133p53α. The expression of MDM2 promotes degradation of the p53 protein, but not Δ133p53α, since Δ133p53α lacks the mdm2-interacting motif. Thus, at a later stage, the accumulation of Δ133p53α not only protects cells from death due to its antiapoptotic function but also provides genetic stability, contributing to the restoration of DNA double stand breaks [89].

It has been reported [90,91] that Δ133p53α plays an important role in embryogenesis and ESC, through sequentially expressing significant levels of Δ133p53α (at least ten-times higher than human fibroblasts). Δ133p53α inhibits FLp53-induced cellular senescence in normal human cells, including fibroblasts, CD8 T lymphocytes and brain astrocytes. An elevated Δ133p53α level plays a causative role in the reprogramming of human cells into a pluripotent state. Induced pluripotent stem cell clones obtained from overexpressing Δ133p53α fibroblasts, when introduced into immunodeficient mice, form well differentiated benign teratomas with differentiation into all tissues of the three germ layers and without malignant pathology. Thus, the activity of *P53* in human pluripotent stem cells is not simply inhibited but is coordinated by Δ133p53α to ensure the creation and maintenance of a self-renewing ability with guaranteed genome stability. The authors conclude that Δ133p53α contributes to many aspects of normal development and healthy lifespan in humans.

Other studies have demonstrated that Δ133p53α protects astrocytes from radiation-induced senescence, promotes DNA repair and inhibits astrocyte-mediated neuroinflammation [92]. In addition, the restoration of Δ133p53α cellular replicating potential may lead to a new therapeutic paradigm for treating immunosensation [93]. Overexpression of Δ133p53α sequentially delays the onset of cell senescence and induces telomerase reverse transcriptase expression, where telomerase activity and is crucial for reprogramming [94], promoting the transformation of human somatic cells into self-renewing pluripotent stem cells with preserved apoptotic and DNA repair activity [95]. Overexpression of Δ133p53α not only increases the efficiency of reprogramming, but also leads to an improvement in the genetic quality in induced pluripotent stem cells [96].

Thus, from the very beginning of our life path, which is nothing more than a direct path to the graveyard, genes/signaling pathways that can self-renew us unlimitedly, work less and less efficiently, and more and more genes/signaling pathways, which are responsible for specialization, differentiation and, ultimately, aging, work more and more. However, it is them who make us what we are – multicellular organisms integrated into a complex hierarchical structure, which are based on tissue-specific differentiation and the basic principle of the adequate functioning of complex multicellular organisms – the subordination of the interests of each unit to the interests of the whole organism. Without these genes, we would be a pile of forever young cells with unlimited proliferative potential and with the ability to turn into anything, anytime and without any regard to the environment. Aside from that, the same genes/factors/signaling pathways allow us to repair DNA damage while preventing apoptosis (p21), to cause apoptosis when repair is not possible (p53) and to carry out many other functions. Furthermore, at a certain stage they contribute to the ‘inclusion’ of the mechanism of reprogramming (rejuvenation). Here again, one can observe the phenomenon of antagonistic bifunctionality. For example, NKX3-1, under normal conditions, is a negative regulator of cell growth and maintains cell differentiated status and its downregulation is associated with the loss of differentiation and tumor growth [97]. Therefore, the function of NKX3-1 is directly opposite to the pluripotency function. However, the expression of the same NKX3-1 precedes the activation of the Yamanaka cell-reprogramming factor Oct4 during reprogramming. Moreover, NKX3-1 replaces exogenous OCT4 to reprogram both mouse and human fibroblasts with comparable efficiency and to generate completely pluripotent stem cells [98].

Another so-called oncogene and Yamanaka factor, *c-Myc*, when overexpressed could lead to opposite effects, for example, the inhibition of proliferative pathways [99] with the restoration of p53 function. However, at a certain stage of expression, p53 activates its own negative regulator Mdm2.

A similar example is the long noncoding RNA gene *PVT1*, which is classified as the so-called oncogene and carries out its function through recruiting of EZH2, stabilization of MDM2 protein expression and restraining *P53* expression, epigenetic regulation of p15 and p16, stimulation of proliferation and inhibition of apoptosis, amplification and stabilization of *Myc*, targeting miR-200a, competing for miR-448 binding to regulate the miRNA target SERBP and inducing epithelial-to-mesenchymal transition [100–107]. This is also confirmed by its amplification in tumors [102,108]. Pvt1a or circular Pvt1 isoforms operate like the so-called oncogenes [102,103,106,109], which include through its interacting with FOXM1 [110]. However, the authors of a recently published paper [111] report that p53-dependent isoform Pvt1b ‘is necessary and sufficient to suppress *Myc* transcription *in cis* without altering the chromatin organization of the locus’. This is consistent with data on tumor-suppressive elements in the Pvt1 locus [109,112,113].

Described above, functional dualism is observed also in embryogenesis. As an example, *ISY1* gene expression during the naive-to-primed ESC transition determines a specific phase of ‘poised’ pluripotency. *ISY1* promotes exit from the naive state and is necessary and sufficient to induce and maintain balanced pluripotency; however, persistent *ISY1* overexpression inhibits the exit from the naive to the primed state [114].

Another example is the antagonistic double effect of phospholipase D2 produced by tumors. Instead of leading to the loss of malignancy through stimulation of cell aging (and this is one of its properties), phospholipase D2 by stimulating aging, leads to the expression of pluripotency factors [115], closing the vicious cycle of cancer and making it impossible to block pluripotency and differentiation accompanied with the loss of malignancy.

**Figure 2.**
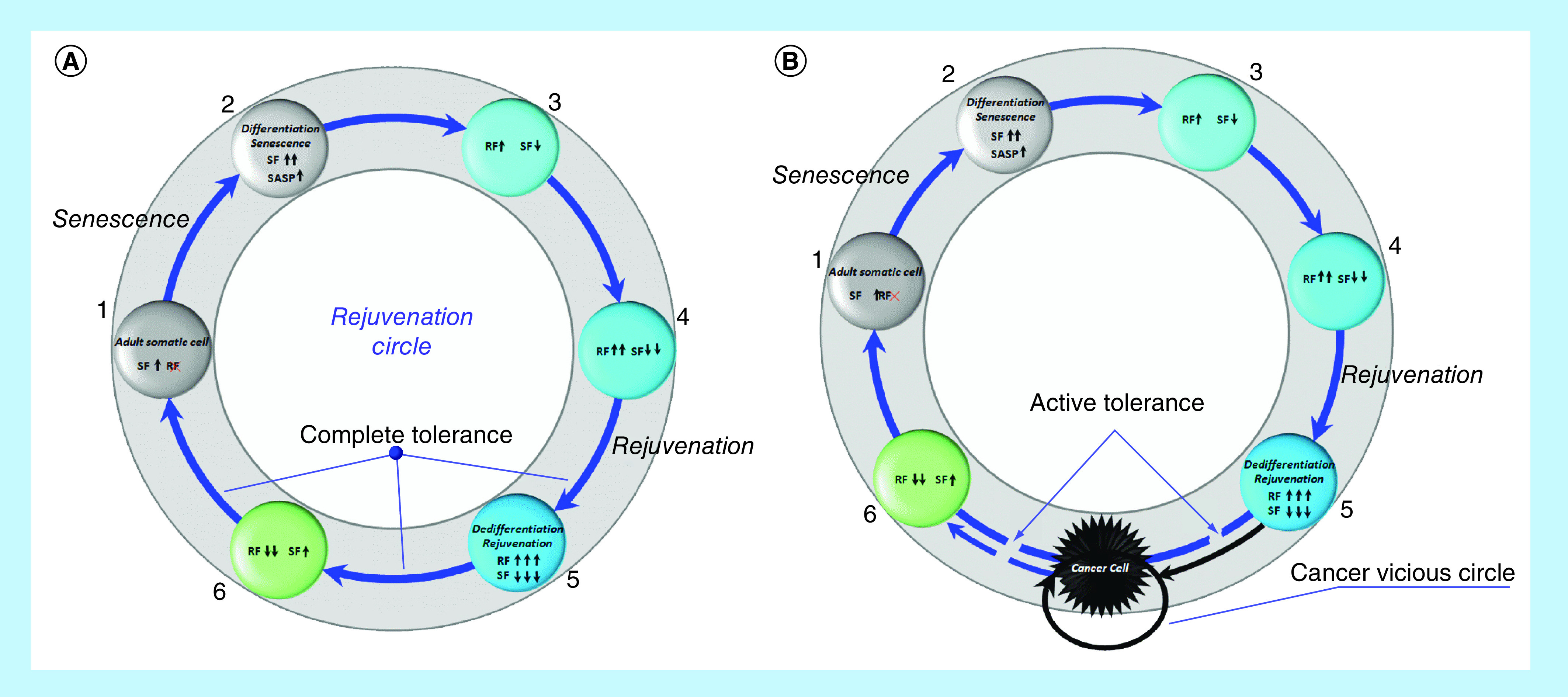
Rejuvenation Circle (RC) or reprogramming (spontaneous or induced) of adult somatic cell under complete immune tolerance conditions (A). Cancer vicious circle (CVC) or reprogramming (spontaneous or induced) of adult somatic cell under active immune tolerance condition **(B)**. RF: Reprogramming/rejuvenation factors, 1–6 – reprogramming steps; SF: Senescence factors.

Under certain conditions, the senescent factors that age us (genes, transcription [translation] factors, signaling pathways of aging), including p53, p63, p73, p15, p21, p27, p130, p16, p14, p18, p19, NKX3-1, Hippo and others, can trigger rejuvenation processes. On the other hand, under certain conditions, the rejuvenation factors that rejuvenate/reprogram/return to pluripotency (genes, transcription [translation] factors, signaling pathways), including Oct4, Sox2, Klf4, c-Myc, Lin28, Nanog, Gata3, Eomes, Tfap2c, Esrrb, Sall4, Ronin, Rae28, Meis1, c-Myb, Cbp, Gata-2, Mll, Bmi-1, c-Ets, c-Jun, MAPK (Ras, Raf) LIF – STAT3, PI3K – Akt, Wnt/b-catenin and others, can inhibit their own activity, contributing to differentiation and prevention of malignant phenotype, make us old. Conversely, what ages us rejuvenates us, and this cell rejuvenation wheel would spin (Figure 2A), resulting in nonaging at the phenotype level, if negative mechanisms of feedback/redifferentiation would not be blocked. As a result of this blocking, instead of the circle of rejuvenation, we switch to the ‘death gear’ of cancer (Figure 2B).

Thus, there are no good and bad genes, isoforms or signaling pathways. All of them are normal genes, isoforms or signaling pathways and carry out normal morphogenetic functions in a determined time and place. The seemingly oncogenic realization of their usual normal functions during the onset of reontogenesis during postnatal development is situationally determined and does not have destructive goals *per se*. Therefore, the task is to create conditions for these genes in its ‘own field’ conditions that mimic immunoprivileged embryogenesis. This is the essence of complete tolerance as a strategy for protecting against cancer with the simultaneous implementation of reontogenesis processes directed toward the rejuvenation and termination of aging.

## How to get postmitotic cells to ‘rebuild themselves’

During ontogenesis, cells such as neurons or myocardiocytes become postmitotic, thus playing an integrative role in the functioning of an organism. The beginning of the ontogenetic program of development includes its own control of division in relation to cells until its complete stop in postmitotic cells, making them one of the main targets for aging processes. To increase the regenerative possibilities of an organism, it is necessary to make postmitotic cells ‘build themselves anew’. The main biological ways to accomplish this is full-scale reprogramming that brings cells back to the early stages of pluripotency. It must be emphasized that what later becomes cancer is initially started as spontaneous reprogramming and the goal is to prevent the transformation of this process into carcinogenesis and direct it as rejuvenation. By creating similar conditions in the body, we can apply safe systemic-induced reprogramming *in vivo*, without fear of resulting in cancer.

According to available data, the possibility of regeneration and reprogramming exists not only for dividing cells, but also for postmitotic cells [116–118]. Presently, there is no data, showing how the continuous reprogramming process can affect neuronal and central nervous system (CNS) functions in general, but there is every reason to believe, that at a low rate of continuous reprogramming there will be no significant violations of the CNS fraction. A not so radical but encouraging way to achieve the processes of rejuvenation in CNS neurons, is the directed influence on their cellular environment, glial cells. According to available data [119,120], glial cells not only participate in neuronal work but, what is especially important, directly affect their metabolism. These data allow us to hope that postmitotic neuronal cells can achieve the effect of ‘division without division’ or metabolic return processes to the state of self-supporting gene activity prevalence.

The proposed concept applied to postmitotic cells is also supported by the data that the reactivation of the classical so-called oncogene (belonging to set of Yamanaka factors) *Myc* and the *CCNT1* gene encoding cyclin T1, which in association with cyclin-dependent kinase 9 (CDK9) plays a key role in carcinogenesis [121], leads to the restoration of the regenerative potential of postmitotic myocardial cells [122]. At the same time, the regenerative transcriptome of postmitotic neurons resulting from damage of the corticospinal tract reverses to an embryonic transcriptional state [123].

In conclusion, we believe it is appropriate to quote Elsasser: “*There are good reasons to suspect that heterogeneity (i.e., variability within any given set of samples) is an essential characteristic of organic life. This idea differs widely from the traditional view that heterogeneity is only a nuisance that is to be circumvented or otherwise eliminated*” [124].

## Future perspective

Thus, we have formulated the concept of looped ontogenesis as a strategy for defeating cancer and aging. A new paradigm has been proposed, and the implementation of which should allow switching spontaneous or induced reontogenesis/remorphogenesis (reprogramming) into the direction of integrative growth (the submission of potency of single cells composing an organism to the development program and functions of the whole organism) while avoiding DG (cancer). For this purpose, it is necessary to model in postnatal ontogenesis a principal condition for normal embryogenesis-immune privilege. The practical implementation of this approach will require two different types of intervention strategies for the early postnatal period (immediately after birth), when the presence of a thymus will allow developing stable central immunological tolerance, and for the later stages of ontogenesis. How all above can be practically accomplished will be presented in Part III of our publication with the subtitle ‘prerequisites and solutions'.

## Definitions

IG – integrating growth, defined here as the submission of potency of single cells composing an organism to the development program and functions of the whole organism

DG – disintegrating growth, defined here as a priority of extension potency of single cells over the development program and functions of the whole organism

**Executive summary**

- Aging is not a special mechanism but a program/order that can be overridden only by implementing another program – a program of permanent, unlimited, quantitatively and qualitatively full restoration of all structures and functional interactions between them and functions. In other words, the linear unidirectionality of ontogenesis, leading to aging and death, can only be overridden with permanent reontogenesis, through the looping process.
- Reontogenesis can be carried out only by cells that are capable of continuous, unlimited, quantitative and qualitative self-renewal. This can be executed only by cells, for which senescence is not the final stage of development but is only an intermediate state, that are capable of constantly ‘zeroing out’ the genetic and metabolic ‘burden’, restoring functional interconnections and functions. There are only two types of cells that are capable of reversing aging, whichsatisfy those criteria: the nonaging totipotent/pluripotent cells of early embryogenesis and the spontaneously reprogrammed cells in precancerous tissues (which then become CSC).
- Targeting one mechanism to revert aging/prevent diseases will not help, but the whole program must be changed. We should find what is the better strategy: stop or help the reontogenesis cycle? Any methods directed against individual mechanisms of ‘aging’ and pathologies associated with age will only ease the burden of aging and associated diseases and slow down their progression but will eliminate neither aging nor disease. By eliminating aging, one will eliminate the aging-related diseases.
- When one attempts to eradicate cancer, one terminates rejuvenation, because spontaneous reprogramming, which then turns into cancer, is an attempt by any living matter to self-renew. Full-scale reprogramming (spontaneous or induced) that never turns into cancer does not have a full-fledged biological alternative for simultaneously solving two main problems: eliminating both cancer and aging. They cannot be solved separately, because the solution to these problems is the same.
- Cancer starts as a spontaneous reprogramming (as a rejuvenation attempt) and only then becomes cancer. Knowing the cause for such a transformation, one will understand its essence and will then be able to direct the processes toward the rejuvenation, preventing their transformation into cancer.
- There are contradictory effects of some of the proteins involved in oncogenesis, gor example, P53, which demonstrates the different functions of them and how they can be/cannot be involved in oncogenesis. There are no good and bad genes, isoforms or signaling pathways. All of them are normal genes, isoforms or signaling pathways and perform normal morphogenetic functions in the right time and place. The seemingly oncogenic implementation of their usual normal functions at the onset of reontogenesis during postnatal development is exclusively situational in nature and does not have destructive goals *per se*.
- Cancer cells in the right environment are not malignant and may be a mechanism of the body to protect from aging.
- Generation of a morphogenetic field in postnatal organs has oncostatic and differentiating properties.
